# The purinergic receptor antagonist oxidized adenosine triphosphate suppresses immune-mediated corneal allograft rejection

**DOI:** 10.1038/s41598-019-44973-y

**Published:** 2019-06-13

**Authors:** William Foulsham, Sharad K. Mittal, Takeshi Nakao, Giulia Coco, Yukako Taketani, Sunil K. Chauhan, Reza Dana

**Affiliations:** 1 Schepens Eye Research Institute, Massachusetts Eye and Ear, Harvard Medical School, Boston, MA USA; 2 Institute of Ophthalmology, University College London, London, UK; 3 Department of Ophthalmology, Osaka University Graduate School of Medicine, Osaka, Japan; 4 Department of Clinical Science and Translational Medicine, University of Rome Tor Vergata, Rome, Italy

**Keywords:** Translational immunology, Allotransplantation

## Abstract

Adenosine triphosphate (ATP) is released into the extracellular environment during transplantation, and acts via purinergic receptors to amplify the alloimmune response. Here, using a well-established murine model of allogeneic corneal transplantation, we investigated the immunomodulatory mechanisms of the purinergic receptor antagonist oxidized ATP (oATP). Corneal transplantation was performed using C57BL/6 donors and BALB/c hosts. oATP or sterile saline was administered via intraperitoneal injection for 2 weeks postoperatively. Frequencies of CD45^+^ leukocytes, CD11b^+^MHCII^+^ antigen presenting cells (APCs), CD4^+^IFN-γ^+^ effector Th1 cells and CD4^+^Foxp3^+^ regulatory T cells (Tregs) were evaluated by flow cytometry. Slit-lamp microscopy was performed weekly for 8 weeks to evaluate graft opacity and determine transplant rejection. Treatment with oATP was shown to significantly reduce graft infiltration of CD45^+^ leukocytes, decrease APC maturation and suppress effector Th1 cell generation relative to saline-treated control. No difference in Treg frequencies or Foxp3 expression was observed between the oATP-treated and control groups. Finally, oATP treatment was shown to reduce graft opacity and increase graft survival. This report demonstrates that oATP limits the alloimmune response by regulating APC maturation and suppressing the generation of alloreactive Th1 immunity.

## Introduction

Keratoplasty is the most common form of allogeneic transplantation worldwide^1^, and remains the definitive treatment for many end-stage corneal diseases^2^. Graft survival rates exceed 90% in recipients with uninflamed and nonvascularized host beds^3^, largely due to the cornea’s particularly tolerant immune microenvironment^4^. Nevertheless, immune rejection remains the primary cause of graft failure^5^, and is a major threat following transplantation onto inflamed or vascularized host beds, where survival rates fall below 50% despite maximal immune suppression^2,6,7^.

The allogeneic response is determined by a complex interplay between pro-inflammatory mechanisms that drive immune rejection, and immunoregulatory pathways that promote tolerance^8^. Following corneal transplantation, there is upregulated local expression of proinflammatory cytokines, chemokines and adhesion molecules^8^. This inflammatory milieu triggers the activation of resident and infiltrating host antigen-presenting cells (APCs), which acquire high levels of major histocompatibility complex class II (MHCII) and other costimulatory molecules^9,10^. Mature APCs recruited from the cornea migrate to the draining lymph nodes (DLN), where they present antigens to naïve T cells, resulting in clonal expansion and differentiation into pro-inflammatory IFNγ-secreting CD4^+^ Th1 cells^8^. Regulatory T cells (Tregs) play a critical role in suppressing the alloimmune response by: (i) modulating APC function, (ii) releasing immunoregulatory soluble factors, (iii) metabolic competition and (iv) direct cytolysis of Th1 cells^11,12^.

By binding to excitatory adenosine triphosphate (ATP) receptors, extracellular ATP amplifies the pro-inflammatory alloimmune response^13,14^. Indeed, extracellular ATP activates purinergic P2X and P2Y receptors to upregulate inflammasome activation in APCs, including dendritic cells and macrophages^13,15,16^. Various events during allotransplantation promote the release of ATP into the extracellular environment, including perioperative trauma, tissue ischemia/reperfusion and immune cell activation^17^. ATP is released to the extracellular environment by stressed, apoptotic or necrotic cells^18,19^. Previous studies have investigated the blockade of ATP using periodate-oxidized ATP (oATP) as a strategy to promote allograft survival in murine models of heart, lung and islet transplantation^20–22^. Other studies have reported the immunomodulatory properties of oATP in models of ischemic tissue damage^23^ and autoimmunity^24–26^. However, there is controversy between these studies about whether the immunosuppressive effects of oATP are primarily mediated via the effector^20,22,26^ or regulatory arms^23^ of the immune response, or indeed both^21,24,25^.

The purpose of this study was to investigate, using a well-established murine model of corneal transplantation^27–30^, the immunomodulatory mechanisms of oATP. Due to the scarcity of resident immune cells in the cornea, this model provides an excellent *in vivo* system to examine the recruitment and function of immune cells. Furthermore, we sought to evaluate oATP as a therapeutic intervention to improve corneal allograft survival. Our data demonstrate that treatment with oATP suppresses APC maturation, limits CD4^+^ effector T cell generation and graft infiltration, and increases corneal allograft survival.

## Results

### Inhibition of the purinergic pathway with oATP reduces corneal infiltration of leukocytes and suppresses APC maturation

**Figure 1:**
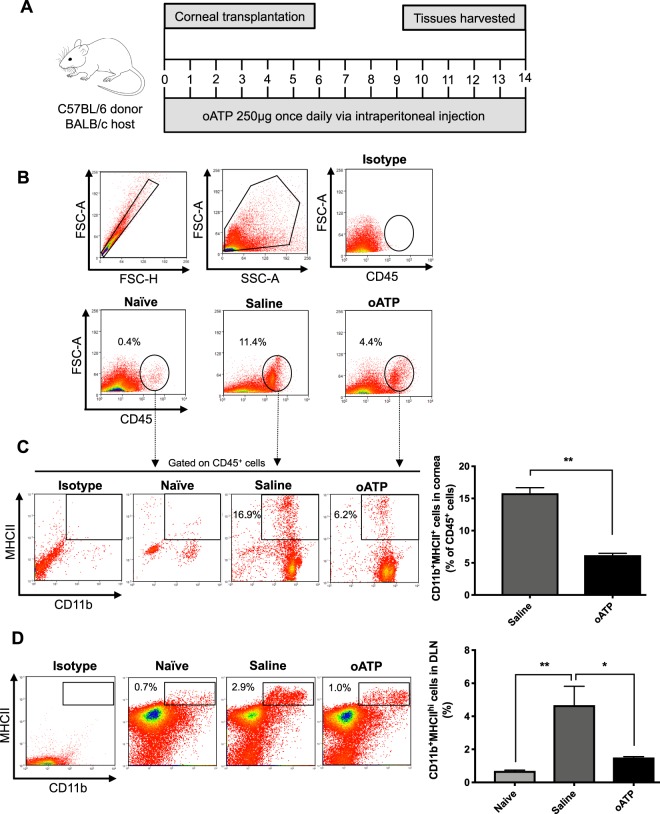
Inhibition of the purinergic pathway with oATP reduces corneal infiltration of leukocytes and suppresses APC maturation. (**A**) Schematic diagram depicting the time points of corneal transplantation, oATP administration and tissue harvesting. (**B**) Representative flow cytometric dot plots showing the gating strategy for, and frequencies of, CD45^+^ inflammatory cells in the corneas of oATP-treated mice, relative to saline-treated and naïve mice. (**C**) Representative flow cytometric dot plots (left) showing the gating strategy for selecting CD11b^+^MHCII^+^ cells in the cornea. Bar chart (right) summarizes the frequencies of CD11b^+^MHCII^+^ cells in the corneas of oATP-treated mice at 14 days after transplantation, relative to saline-treated mice. **(D)** Representative flow cytometric dot plots (left) and bar chart (right) depicting the frequencies of CD11b^+^MHCII^hi^ cells in the draining lymph nodes of oATP-treated mice, relative to saline-treated and naïve mice. n = 5–7/group. Representative data from three independent experiments are shown, and data are depicted as mean ± SEM. *p < 0.05; **p < 0.01.

To investigate the effect of treatment with oATP on corneal infiltration of immune cells, graft recipients received intraperitoneal injection of oATP postoperatively and tissues were collected (Fig. 1A). Saline-treated animals served as controls. Flow cytometric data demonstrated an increase in corneal infiltration of CD45^+^ leukocytes in saline-treated animals, which was substantially reduced in the oATP-treated group (Fig. 1B). Similarly, the frequencies of MHCII^+^CD11b^+^ mature APCs were significantly reduced in the oATP-treated group following transplantation, as compared to the saline-treated group (Fig. 1C). Having observed that treatment with oATP resulted in lower frequencies of mature APCs at the graft site, we investigated whether oATP also inhibited maturation of APCs in the DLNs. Our data demonstrate that treatment with oATP abrogates the increased frequencies of CD11b^+^MHCII^+^ cells in the DLNs of saline-treated mice following transplantation (Fig. 1D).

### Treatment with oATP suppresses CD4^+^ effector T cell generation and graft infiltration

**Figure 2:**
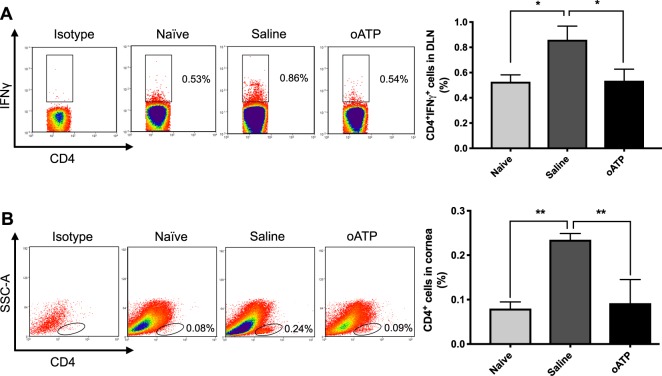
Treatment with oATP suppresses CD4^+^ effector T cell generation and graft infiltration. (**A**) Representative flow cytometric dot plots (left) and cumulative bar chart (right) showing the frequencies of CD4^+^IFNγ^+^ cells (gated on CD4^+^ cells) in the draining lymph nodes of oATP-treated mice, compared to saline-treated and naïve mice. **(B**) Representative flow cytometric dot plots (left) showing the gating strategy for selecting CD4^+^ cells in the cornea. Bar chart (right) summarizes the frequencies of CD4^+^ cells in the cornea at 2 weeks post-transplantation, relative to saline-treated and naïve mice. n = 5–7/group. Representative data from three independent experiments are shown, and data are depicted as mean ± SEM. *p < 0.05; **p < 0.01.

During the alloimmune response, the inflammatory microenvironment at the cornea results in the acquisition of MHCII and co-stimulatory molecules by local APCs^8^. These mature APCs egress from the graft site via lymphatic vessels to the DLNs, where they prime naïve T cells by presenting alloantigens, resulting in the expansion and differentiation of a population of IFN-γ–secreting CD4^+^ Th1 cells^8^. In our study, we sought to establish whether the decreased frequencies of CD11b^+^MHCII^+^ APCs observed in the DLNs of oATP-treated mice resulted in decreased frequencies of effector T cells. Consistent with our previous publications, we observed a significant increase in CD4^+^IFN-γ^+^ Th1 cells in the DLNs following transplantation in the saline-treated group^27,31^. Notably, this phenomenon was abrogated in the oATP-treated hosts (Fig. 2A). Indeed, evaluation of CD4^+^ cell infiltration in the cornea revealed a similar pattern, with amplified frequencies in the saline-treated group compared to naïve mice, but with oATP treatment decreasing the CD4^+^ cell frequencies to baseline (Fig. 2B). Having observed that treatment with oATP both reduced frequencies of mature APCs and IFN-γ–secreting CD4^+^ Th1 cells following corneal transplantation, we examined whether oATP had a *direct* effect on T cells, or whether the decreased frequencies of Th1 cells observed were secondary to the inhibitory effect of oATP on APCs. Our *in vitro* mixed lymphocyte reactions using APCs derived from C57BL/6 mice and T cells from BALB/c mice indicate that, in addition to its APC-mediated immunoregulatory activity, oATP has a direct effect on alloreactive T cells. Indeed, treatment of T cells with oATP resulted in a 34% reduction in dilution of CFSE-labeled cells, compared with a 64% reduction when APCs were treated with oATP, and a 74% reduction when both T cells and oATP were treated with oATP (Fig. S1).

### oATP treatment does not promote the significant expansion of regulatory T cell frequencies following corneal transplantation

**Figure 3:**
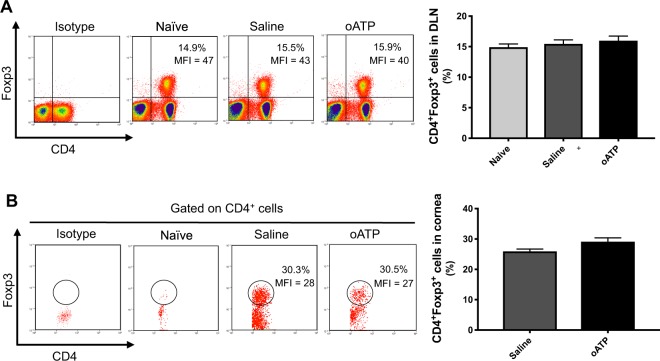
oATP treatment does not promote the significant expansion of regulatory T cell frequencies following corneal transplantation. (**A**) Representative flow cytometric dot plots (left; with Foxp3 MFI) and cumulative bar chart (right) showing the frequencies of CD4^+^Foxp3^+^ cells in the draining lymphoid tissue of oATP-treated recipients, saline-treated recipients and naïve mice. (**B**) Flow cytometric dot plots demonstrating the gating strategy for enumerating CD4^+^Foxp3^+^ cells (gated on CD4^+^ cells) in the cornea (left), with protein expression (median fluorescence intensity; MFI) of Foxp3 detailed. Bar chart (right) summarizes the frequencies of corneal CD4^+^Foxp3^+^ cells at day 14 post-transplantation in oATP-treated and saline-treated recipients, relative to naïve mice. n = 5–7/group. Representative data from three independent experiments are shown, and data are depicted as mean ± SEM.

In order to evaluate the effect of oATP treatment on Tregs, we harvested DLNs following transplantation and analyzed single cell suspensions by flow cytometry (Fig. 1A). No difference in the frequencies of CD4^+^Foxp3^+^ Tregs was detected between naïve mice, saline-treated allograft recipients and oATP-treated allograft recipients (Fig. 3A). Moreover, Foxp3 expression levels were similar between the saline- and oATP-treated hosts (median fluorescence intensity [MFI] equal to 43 and 40 respectively). As with the lymph node data, corneal single cell suspensions derived from saline- and oATP-treated groups showed similar frequencies of CD4^+^Foxp3^+^ cells and levels of Foxp3 expression (MFI equal to 28 and 27 respectively; Fig. 3B). The functional relevance of Foxp3 expression as a marker of Treg suppressive activity in transplantation has been demonstrated in previous work by our laboratory^12^. Nevertheless, we conducted additional *in vitro* Treg functional assays, in which treatment of Tregs with oATP was not observed to result in a substantial reduction in T cell proliferation as compared to PBS-treated Tregs (Fig. S2).

### Treatment with oATP decreases graft opacity and promotes corneal allograft survival

**Figure 4:**
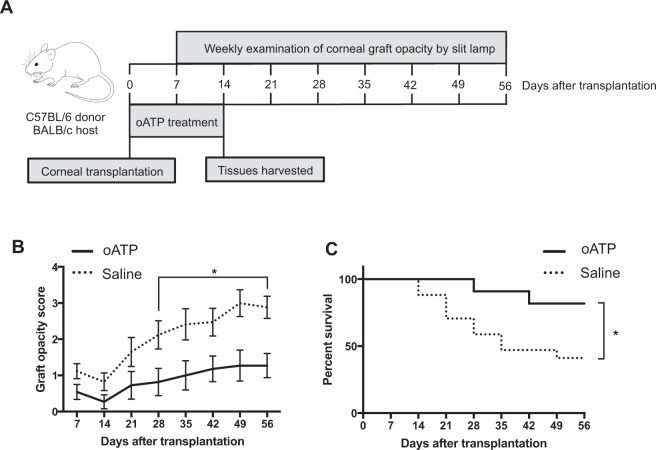
Treatment with oATP decreases graft opacity and promotes corneal allograft survival. Allogeneic corneal transplantation was performed and oATP (10 μg/g mouse body weight) or saline (control) were administered via intraperitoneal injection once daily for 14 days starting from the day of transplantation. Graft opacity was evaluated for eight weeks following transplantation, and graft survival was determined according to an established scoring system. **(A)** Schematic diagram depicting the time points of slit lamp examination of corneal graft opacity. (**B**) Graft opacity scores were significantly decreased in the oATP-treated group from week 4 until week 8 (p < 0.05). **(C)** Kaplan-Meier survival curve of oATP-treated graft recipients vs. saline-treated controls. Logrank test demonstrates significantly improved survival in the oATP-treated group relative to control (p = 0.032). Data shown is pooled from two independent experiments (n = 6–10/group). Data is represented as mean ± SEM.

Finally, we investigated the effect of oATP treatment on graft opacity and transplant survival. Graft opacity was assessed by slit lamp microscopy once weekly for eight weeks (as depicted in Fig. 4A), and graft survival was determined as previously described^27–30^. The oATP-treated transplanted group exhibited significantly lower graft opacity scores compared to the saline-treated group from postoperative week 4 onwards (Fig. 4B). A concomitant increase in the survival of the grafts in oATP-treated recipients, relative to saline-treated controls, was observed (Fig. 4C).

## Discussion

The activation of purinergic P2X and P2Y receptors by extracellular ATP amplifies inflammation^13,15,16^. This study advances our understanding of the immunoregulatory capacities of periodate-oxidized ATP, an irreversible antagonist of ATP, in the setting of corneal allotransplantation. Specifically, our data show that following corneal grafting, treatment with oATP: (1) reduces corneal infiltration of leukocytes and suppresses APC maturation; (2) decreases frequencies of Th1 cells in draining lymphoid tissue, as well as graft-infiltrating CD4^+^ T cells; and (3) increases corneal transplant survival.

Tissue damage resulting from allotransplantation triggers the release of ATP either from necrotic cells or via pannexin channels in activated immune cells and apoptotic cells^17^. APCs are known to express purinergic receptors, including P2X_7_R and P2Y_2_R, that when activated upregulate the expression of MHCII and co-stimulatory molecules^14^. In our study, we demonstrate that blockade of the purinergic pathway with oATP results in a reduction of mature APCs at both the graft site and draining lymphoid tissues (Fig. 1C,D). Notably, the oATP-mediated suppression of APC maturation in the setting of allotransplantation has not previously been reported^20–22^. However, in a model of renal ischemia-reperfusion injury, Koo and colleagues have demonstrated that systemic treatment with oATP prior to ischemia-reperfusion injury results in decreased frequencies of mature APCs in the kidney compared to saline-treated controls^23^. By suppressing APC maturation in corneal allotransplantation, oATP limits the priming of naïve T cells.

After priming by APCs, naïve T cells differentiate into CD4^+^IFNγ^+^ Th1 effectors, the principal mediators of acute corneal graft rejection^8,10^. Our data demonstrate a significant increase in the frequencies of CD4^+^IFNγ^+^ cells in the DLNs of saline-treated allograft recipients, but this effect is fully abrogated by treatment with oATP (Fig. 2A). In a study using a murine model of cardiac transplantation, Vergani and colleagues reported that treatment with oATP promotes cardiac transplant survival in 80% of murine recipients of a fully mismatched allograft (C57BL/6 recipients of BALB/c donor hearts)^21^. Furthermore, the authors describe reduced numbers of IFN-γ-producing cells in spleens harvested from oATP-treated mice compared to untreated controls, with a difference between the groups that was maintained at day 100 post-transplantation^21^. Similar findings have been reported in a murine model of islet allograft rejection^20^. These studies are consistent with our data, suggesting that treatment with oATP suppresses the Th1 alloimmune response. It is interesting to note that in Vergani and colleagues’ cardiac transplant study^21^, as well as in other studies of lung^22^ and islet transplantation^20^, short-term systemic treatment with oATP was demonstrated to improve long-term graft survival. We also observed that 14 days’ treatment with oATP promoted transplant survival at 8 weeks, suggesting that by suppressing the acute phase of the alloimmune response, oATP has enduring effects on allograft survival.

There has been some controversy in the literature regarding the effect of systemic oATP on Tregs. Liu and colleagues report significantly reduced numbers of Tregs in lung allografts from oATP-treated animals as compared to vehicle controls^22^. In contrast, using a model of renal ischemia-reperfusion injury, Koo and colleagues attribute the therapeutic function of oATP to the expansion of regulatory T cells^23^. Here, the investigators observed frequencies of CD4^+^Foxp3^+^ T cells in the kidneys of oATP-treated mice more than four times greater than those detected in the vehicle control group. However, in a mouse model of cardiac transplantation, Vergani and colleagues report no difference in peripheral Treg frequencies between oATP-treated animals and vehicle controls, but observed a significant increase in Treg frequencies in oATP-treated animals at 100 days post-transplantation^21^. Moreover, a study conducted by the same group on islet transplantation demonstrated no difference between oATP-treated animals and vehicle controls following transplantation^20^.

Given the critical role of Tregs in limiting the alloimmune response in corneal transplantation^11,12^, in our study we investigated the effect of systemic oATP treatment on Treg frequencies both at the cornea and draining lymphoid tissues. Our data show no significant difference CD4^+^Foxp3^+^ Treg frequencies either at the graft site or DLNs (Fig. 3). Foxp3 expression is known to reflect the functional status of Tregs in allotransplantation^12^. However, our data do not reveal a significant difference in Foxp3 expression (MFI) between oATP-treated and saline-treated animals.

The purpose of this study was not to conduct a comprehensive survey of immune cells modulated by oATP, and it is conceivable that oATP promotes graft survival by suppressing a variety of different pro-inflammatory cells. Indeed, previous studies have demonstrated a direct effect of oATP on T cells (i.e. independent of APCs), which may partially account for the decreased frequencies of pathogenic T cells and improved allograft survival observed in our study^24,26^. Yet the work of our group^29,32,33^ and others^31,34–37^ has established that in our murine model of corneal transplantation, the activation of CD11b^+^MHCII^+^ cells and subsequent infiltration of graft-attacking CD4^+^ T cells plays a critical role in determining immune rejection. Our previous studies have established that 14 days post-transplantation is an optimum time point to evaluate the alloimmune response prior to the onset of graft rejection^38–40^, yet it is feasible that kinetic analysis of immune cell frequencies and activation may have captured additional immunomodulatory effects of oATP. Indeed, we have observed substantial suppression of APC maturation as early as day 3 post-transplantation in mice treated with oATP compared to PBS control (Fig. S3).

Our data evaluating graft survival demonstrate that treatment with oATP significantly reduces corneal graft opacity and promotes transplant survival compared to saline-treated controls (Fig. 4). Taken together, our findings strongly suggest that oATP promotes allograft survival by suppressing the effector arm of the alloimmune response. Moreover, our data imply that limiting the Th1 response with oATP treatment may be a viable therapeutic strategy to promote the survival of corneal allografts.

## Methods

### Animals

Six- to eight-week old male C57BL/6 (donors) and BALB/c (hosts) mice were purchased from Charles River Laboratories (Wilmington, MA). Animals were housed in a secure, pathogen-free environment at the Schepens Eye Research Institute Animal Facility. Animals were treated in accordance with the guidelines set forth by the Association for Research in Vision and Ophthalmology (ARVO) Statement for the Use of Animals in Ophthalmic and Visual Research. All experiments were reviewed and approved by the Schepens Eye Research Institute Animal Care and Use Committee.

### Corneal transplantation

Allogeneic orthotopic corneal transplantation was performed as described previously^27–30^. Briefly, 2 mm diameter donor buttons were excised from the central cornea of C57BL/6 mice using scissors (Vannas; Storz Instruments, San Dimas, CA). BALB/c host beds were prepared by excising a 1.5 mm diameter button from the central cornea. The donor button was then secured into position on the recipient bed via 8 interrupted 11–0 nylon sutures (AB-0550S, MANI, Tochigi, Japan). Tarsorrhaphy was performed with a single 8–0 suture (AB-2056, MANI, Tochigi, Japan) to close the eyelid until post-operative day 3. Corneal sutures were removed at post-operative day 7. The surgical procedures were performed using intraperitoneal ketamine (120 mg/kg bodyweight) and xylazine (20 mg/kg bodyweight) for anesthesia. Furthermore, buprenorphine (0.1 mg/kg bodyweight) was administered intraoperatively and every 8–12 hours postoperatively (up to 48 hours) as analgesia.

### Evaluation of graft survival

Graft survival was assessed for 8 weeks using a slit-lamp biomicroscope. Graft opacity was scored on a weekly basis according to an established scoring system (range, 0–5+); with immune rejection defined by a score of 2+ for two consecutive examinations (i.e. a level of opacity that obscures iris details)^27–30,41^. To exclude grafts undergoing primary failure, those grafts with scores 2+ at post-operative day 14 were excluded from experimentation and analysis.

### oATP treatment protocol

oATP (250 μg, Sigma Aldrich, St. Louis, MO) diluted in 100 μl sterile saline was administered via intraperitoneal (i.p.) injection once daily for the 14 days following grafting (Fig. 1A), based on the treatment schedule in previously published transplantation studies^20–22^. The first dose of oATP was administered intraoperatively, at the time of transplantation. The control group was administered daily i.p. injections of 100 μl sterile saline.

### Isolation of corneal cells and lymph node cell preparation

Collagenase digestion was used to prepare single-cell suspensions from the corneal samples, as described previously^27,42^. Briefly, corneas were digested in RPMI media (Lonza, Walkersville, MD) containing 2 mg/ml DNase I (Roche, Basel, Switzerland) and 2 mg/ml collagenase type IV (Sigma-Aldrich, St. Louis, MO) for 60 min at 37 °C. Following this, samples were filtered through a 70-μm cell strainer. DLNs in the submandibular and cervical regions were harvested and single cell suspensions were prepared, as described previously^27^.

### Flow cytometry

Single cell suspensions were stained with fluorochrome-conjugated antibodies directed against cell surface expression CD11b and MHCII on APCs, CD45 on inflammatory cells, as well as CD4 on T cells. For surface staining, cells were incubated with monoclonal antibodies or appropriate isotype controls for 30 minutes in the dark, on ice. Following this, cells were washed twice using PBS. Stained cells were suspended in PBS, and analyzed using a LSR II flow cytometer (BD Biosciences, San Jose, CA) and Summit v4.3 software (Beckman Coulter, Indianapolis, IN). Dead cells were excluded, doublet discrimination was performed by gating simultaneously on FSC_height_ vs. FSC_area_ and appropriate Fluorescence Minus One (FMO) controls were used. Antibodies and matched isotype controls were obtained from Biolegend, San Diego, CA. Stimulation with phorbol 12-myristate 13-acetate (PMA; 20 ng/ml; Sigma-Aldrich, St. Louis, MO) and ionomycin (1 ug/ml; Sigma-Aldrich, St. Louis, MO) for 4 hours at 37 °c in the presence of Golgistop (0.1 µl/100 µl media; BD Bioscience) was performed prior to intracellular evaluation of IFN-γ in type 1 T helper cells (Th1), as previously described^43^. Intracellular staining was performed in order to evaluate the expression of interferon-γ (IFN-γ) in Th1 cells and forkhead box P3 (Foxp3) in CD4^+^ cells. Following cell surface staining, cells were washed twice with PBS and resuspended in Fixation/Permeabilization Solution (Thermo Fischer, Waltham, MA) for 60 minutes at 4 °C. Cells were subsequently washed with Perm/Wash Buffer (Thermo Fischer, Waltham, MA) and stained with fluorochrome-conjugated anti-IFN-γ or anti-Foxp3, or matched isotype antibody controls, for 45 minutes on ice. Antibodies and matched isotype controls and were obtained from Biolegend, San Diego, CA. Finally, cells were washed and suspended in PBS before analysis using a LSR II flow cytometer (BD Biosciences, San Jose, CA) and Summit v4.3 software (Beckman Coulter Life Sciences, Indianapolis, IN).

### Statistical analysis

Mann-Whitney U tests or unpaired two-tailed Student’s t-tests were used to compare groups, as determined by Shapiro-Wilk test^44^. The Kaplan-Meier survival curve was used to determine graft survival, and the logrank test was used to compare survival rates between the groups. p < 0.05 was considered statistically significant. Data are presented as the mean ± standard error (SEM). Results shown are representative of three independent experiments. Samples sizes were estimated based on previous experimental studies on corneal transplantation^29,30,39,45,46^.

## Supplementary information

Supplementary Figures

## Data Availability

The datasets generated during and/or analysed during the current study are available from the corresponding author on reasonable request.
