# Supplementary material for: The purinergic receptor antagonist oxidized adenosine triphosphate suppresses immune-mediated corneal allograft rejection
Source: Sci Rep. 2019 Jun 13;9:8617. doi: 10.1038/s41598-019-44973-y (PMC6565802; doi:10.1038/s41598-019-44973-y)
Supplement: Supplementary file 1 — Supplementary Figures [file 41598_2019_44973_MOESM1_ESM.pdf]

The purinergic receptor antagonist oxidized adenosine triphosphate suppresses immune-mediated corneal allograft rejection

William Foulsham (MBChB)[1+], Sharad K. Mittal (Ph.D.)[1+], Takeshi Nakao (M.D.)[1,2], Giulia Coco (M.D.)[1,3], Yukako Taketani (M.D., Ph.D.)[1], Sunil K. Chauhan (D.V.M., Ph.D.)[1], Reza Dana (M.D., M.Sc., MPH)[1*]

[+] Co-first authors

* Corresponding authors

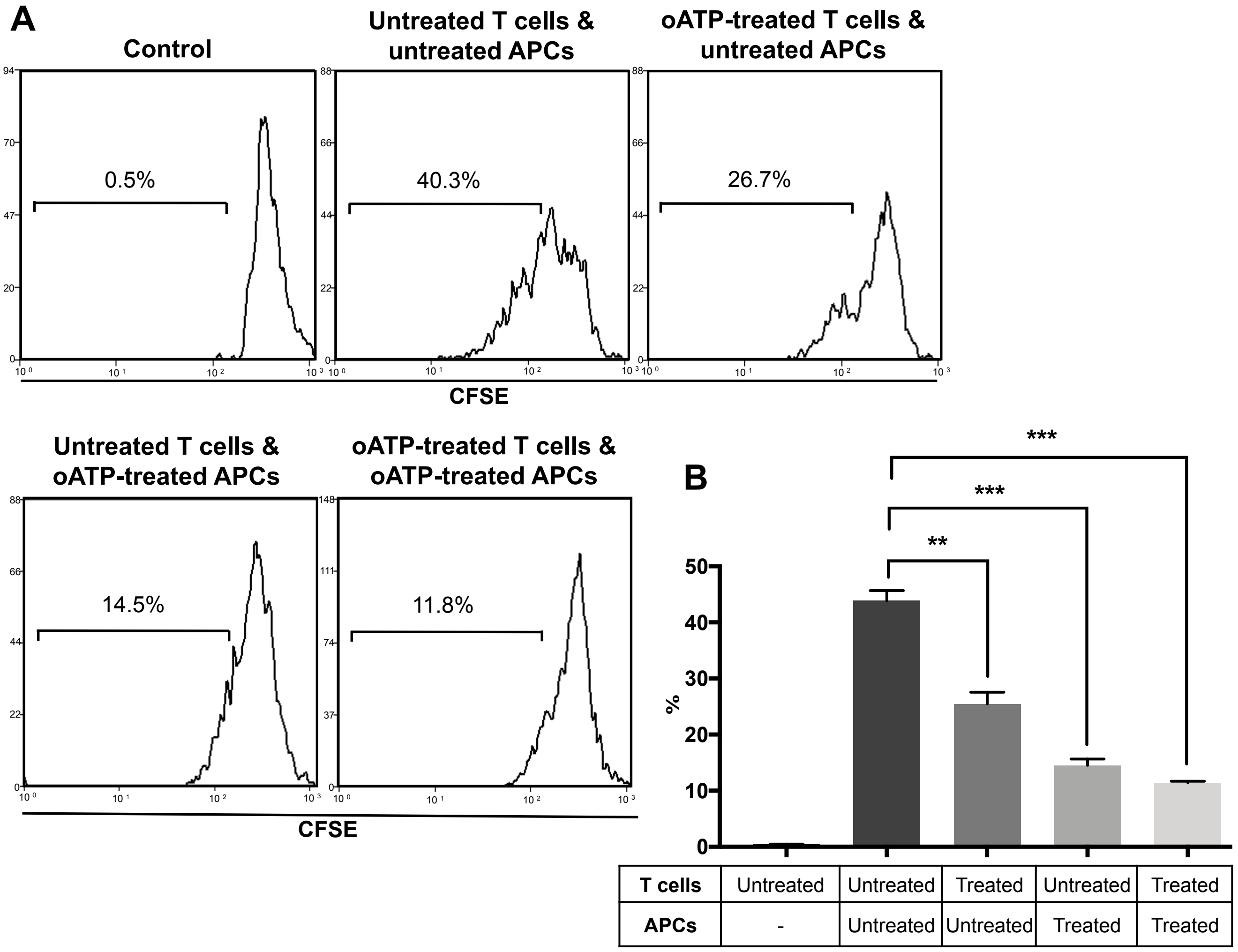

Supplementary Figure 1. oATP directly suppresses antigen presenting cells and T cells in mixed lymphocyte reaction. T cells ($CD90.2^{+}$) from BALB/c mice and APCs ($CD90.2^{-}$) from C57BL/6 mice were purified from spleens and lymph nodes by magnetic sorting. Isolated T cells were labeled with CFSE (5 μM; Invitrogen). Following this, oATP (500 μM; Sigma Aldrich)-treated or PBS-treated T cells were co-cultured with oATP-treated or PBS-treated APCs in the media containing exogenous ATP (100 nM) for 5 days. Histograms (top and left) and bar chart (bottom right) depicting the proliferation of CFSE-labeled T cells in MLR cultures following indicated treatments are shown. Experiments were repeated three times, and data in each group are from triplicate wells. Data are depicted as mean ± SEM. **p<0.01; ***p<0.001.

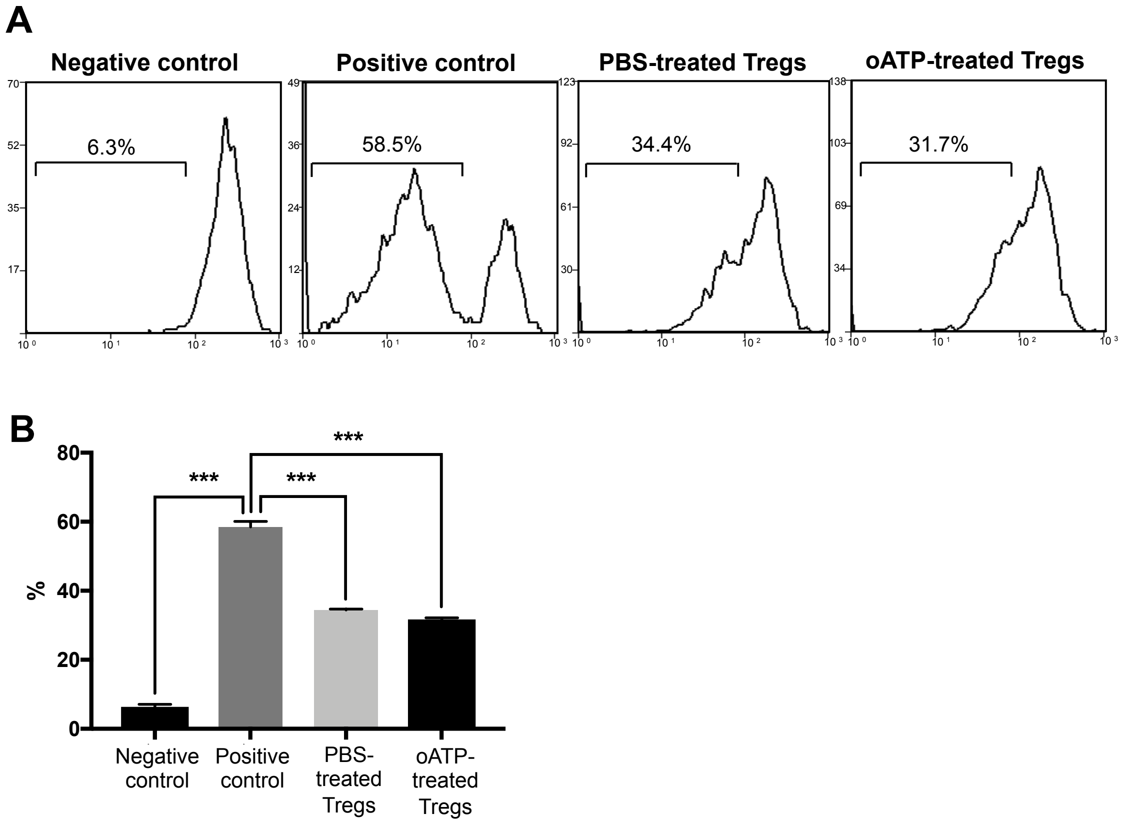

Supplementary Figure 2. oATP treatment does not substantially modulate Treg suppressive function. $CD4^+CD25^-$ naïve T cells and $CD4^+CD25^+$ Tregs were isolated from the spleens and lymph nodes of BALB/c mice using magnetic separation kits (Miltenyi Biotec). Naïve T cells (1×10^5) were labeled with CFSE (5 μM; Invitrogen) and co-cultured with oATP (500 μM; Sigma Aldrich)-treated $CD4^+CD25^+$ Tregs (5×10^4), T cell-depleted syngeneic splenocytes (1×10^5) and 1 μg/ml anti-CD3 antibody for 3 days in the presence of exogenous ATP (100nM; Tocris). T cells cultured alone served as negative controls. T cells cultured with syngeneic splenocytes and anti-CD3 served as positive controls. Representative histograms (**A**) and bar chart (**B**) show comparable dilution of CFSE in proliferating T cells cultured with oATP-treated or PBS-treated Tregs. Experiments were repeated three times, and data in each group are from triplicate wells. Data are depicted as mean ± SEM. ***$p<0.001$.

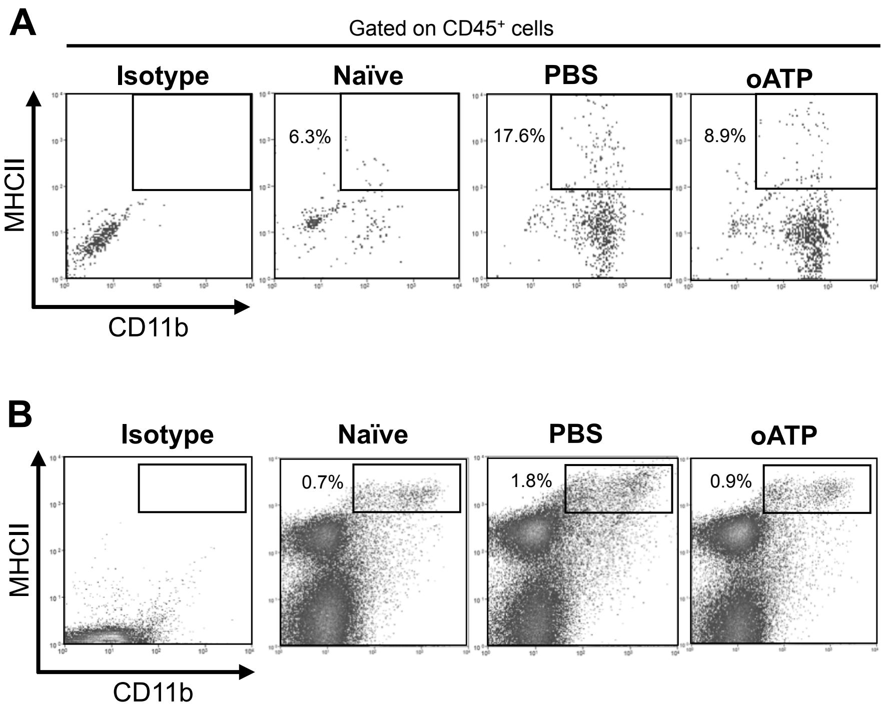

Supplementary Figure 3. oATP administration suppress the early infiltration of mature APCs in graft recipients. At day 3 post-transplantation, corneas and draining lymph nodes were harvested from oATP-and PBS-treated graft recipients. Flow cytometry was performed on single cell suspensions of harvested tissues. **(A)** Representative flow plots showing reduced infiltration of mature $CD11b^{+}MHCII^{+}$ antigen presenting cells (gated within $CD45^{+}$ cells) in the corneas of oATP-treated graft recipients compared to PBS-treated groups. **(B)** FACS plots showing diminished frequencies of $CD11b^{+}MHCII^{+}$ APCs in the draining lymph nodes of graft recipients following oATP administration. n = 4/group. Representative data from two independent experiments are shown.
